# CAD/CAM for scalable nanomanufacturing: A network-based system for hybrid 3D printing

**DOI:** 10.1038/micronano.2017.72

**Published:** 2017-09-25

**Authors:** Hae-Sung Yoon, Hyun-Taek Lee, Ki-Hwan Jang, Chung-Soo Kim, Hyunseo Park, Dae-Wook Kim, Kunwoo Lee, Sangkee Min, Sung-Hoon Ahn

**Affiliations:** 1 BK21 Plus Transformative Training Program for Creative Mechanical and Aerospace Engineers, Seoul National University, Seoul 08826, Republic of Korea; 2 Department of Mechanical and Aerospace Engineering, Seoul National University, Seoul 08826, Republic of Korea; 3 Department of Mechanical Engineering, University of Wisconsin-Madison, Madison, WI 53706, USA; 4 Institute of Advanced Machines and Design, Seoul National University, Seoul 08826, Republic of Korea

**Keywords:** Computer-aided design and manufacturing (CAD/CAM), nanomanufacturing, network-based, 3D printing

## Abstract

Micro- and nano-structuring have been highlighted over several decades in both science and engineering fields. In addition to continuous efforts in fabrication techniques, investigations in scalable nanomanufacturing have been pursued to achieve reduced feature size, fewer constraints in terms of materials and dimensional complexity, as well as improved process throughput. In this study, based on recent micro-/nanoscale fabrication processes, characteristics and key requirements for computer-aided design and manufacturing (CAD/CAM) systems for scalable nanomanufacturing were investigated. Requirements include a process knowledge database, standardized processing, active communication, adaptive interpolation, a consistent coordinate system, and management of peripheral devices. For scalable nanomanufacturing, it is important to consider the flexibility and expandability of each process, because hybrid and bridging processes represent effective ways to expand process capabilities. As an example, we describe a novel CAD/CAM system for hybrid three-dimensional (3D) printing at the nanoscale. This novel hybrid process was developed by bridging aerodynamically focused nanoparticle printing, focused ion beam milling, micromachining, and spin-coating processes. The system developed can print a full 3D structure using various inorganic materials, with a minimum process scale of 50 nm. The most obvious difference versus CAD/CAM at ‘conventional’ scales is that our system was developed based on a network to promote communication between users and process operators. With the network-based system, it is also possible to narrow the gap among different processes/resources. We anticipate that this approach can contribute to the development of CAD/CAM for scalable nanomanufacturing and a wide range of hybrid processes.

## Introduction

Micro- and nano-structuring have been highlighted in recent decades due to their unique properties such as higher density of functionality. Recent developments in manufacturing include new fabrication techniques in fields including optics^1,2^, communications^3,4^, engineering surfaces^5,6^, metamaterials^7^, and sensors^8^. In addition to the development of novel fabrication techniques, ‘conventional’ manufacturing processes, such as mechanical machining, have continued to evolve to achieve nanoscale capabilities in terms of process scale and precision^9,10^.

Developments in micro-/nanoelectromechanical system (MEMS/NEMS) technologies have contributed to various applications^11^, and the semiconductor-related technologies of patterning, deposition, and etching have enabled the mass production of micro-/nanodevices^12^. With batch fabrication, cost per chip can be reduced significantly^13^. Recent advances in MEMS/NEMS research has sought to reach beyond the fundamental limits of previous processes^14,15^ in terms of process scale^16^, material selectivity^17^, and geometric complexity^18^. Some technologies use polymers and hydrogels to even deal with structures that change motions^19^. In addition to semiconductor techniques, various fabrication processes have been investigated to overcome existing process barriers.

Hybrid and bridging processes represent an effective way to not only enhance process capabilities but also develop novel processes with synergies beyond the capabilities of the individual processes^20,21^. For example, many semiconductor technologies can be regarded as hybrid processes, such as integrating additive (lithography) and subtractive processes (etching). Researchers have attempted to bridge the gaps among various processes by integrating top–down and bottom–up processes^22,23^.

However, despite the continued effort and advances, the available technologies are not yet fully applicable to manufacturing at the nanoscale to realize scalable, high-yield processes with high degrees of freedom. Achieving mass manufacturability and high flexibility has been a major issue in manufacturing generally, and is much more challenging at the nanoscale^15,21^. Indeed, because we are reaching the fundamental limits of individual fabrication processes, it is important to seek synergies among them for ‘scalable nanomanufacturing’ with mass manufacturability and high flexibility.

Moreover, research on computer-aided design and manufacturing (CAD/CAM) systems is necessary for scalable nanomanufacturing processes. Software is available for the simulation and design of MEMS devices in various engineering fields^13^; however, consensus on CAD/CAM for scalable nanomanufacturing has yet to be achieved, and many challenges remain in nanomanufacturing. Particularly for hybrid processes, it is more important to integrate and manage individual processes from the software perspective. Given this background, it is important to review the characteristics of recent micro-/nanomanufacturing (MNM) processes and identify the essential requirements of CAD/CAM systems for scalable nanomanufacturing.

In this research, CAD/CAM systems for scalable nanomanufacturing were investigated based on the characteristics of micro-/nanomanufacturing systems. Here we compare the characteristics of micro-/nanomanufacturing with those of conventional fabrication techniques, and also present a scenario for a nanoscale three-dimensional (3D) printing system. In section “MATERIALS AND METHODS”, recent approaches in micro-/nanomanufacturing are reviewed briefly, as well as the paradigm shift in manufacturing in general, and their characteristics are analyzed. Scope of the term scalable nanomanufacturing is discussed. Then, based on our comparison, characteristics and key requirements for CAD/CAM in scalable nanomanufacturing are suggested in section “Results and Discussion”. Because scalable nanomanufacturing is a developing area, we focused mainly on the flexibility and expandability of individual processes with respect to the CAD/CAM system. As an example, a novel CAD/CAM system for hybrid 3D printing at the nanoscale is described. This hybrid 3D printing system was developed to achieve a reduced feature size, with fewer constraints in terms of materials and dimensional complexity and improved process throughput. The novel hybrid process was developed by bridging aerodynamically focused nanoparticle (AFN) printing and focused ion beam (FIB) milling, micromachining, and spin-coating processes. By integrating different micro-/nanoscale processes, 3D features can be fabricated with a minimum process scale of 50 nm. The CAD/CAM system was constructed on a network-based platform. Designers and operators can freely access and control part geometry and process planning. Characteristics of the CAD/CAM system developed are discussed, and compared with suggested key requirements for CAD/CAM generally for scalable nanomanufacturing processes.

We anticipate that this approach can contribute to the development of a concept for CAD/CAM for scalable nanomanufacturing, as well as CAD/CAM for novel hybrid processes. As with MEMS/NEMS technologies, scalable nanomanufacturing is expected to contribute to unique engineering applications.

## Materials and methods

### Micro-/nanomanufacturing systems

Recent micro-/nanoscale manufacturing systems have adopted various physical/chemical phenomena to fabricate structures. Process capabilities are significantly influenced by the physics involved; many researchers have classified and evaluated processes in terms of process physics or processing format. Razali *et al.*^24^ classified micromanufacturing processes into subtractive, additive, deforming, joining, and hybrid processes. Chu *et al.* reviewed hybrid manufacturing processes on the micro-/nanoscale, and classified technologies in terms of processing format; for example, machining, deposition, drilling, etching, and lithography^25^. Yoon *et al.*^20^ classified micro-/nanoscale fabrication processes in terms of being energy beam-, liquid/aerosol-, or tip-based.

Table 1 shows comparative examples of micro-/nanoscale fabrication techniques in terms of processing format with respect to process scale, resolution, and geometric degrees of freedom. For the brief relative comparison, general characteristics are indicated by comparisons of individual processes, which have clear pros and cons in terms of the process physics used. Meeting the needs for high precision, high geometric degrees of freedom, and high throughput have been major issues in many manufacturing processes. Particularly in micro-/nanomanufacturing, fabrication technologies have been developed with the goal of scalable nanomanufacturing for practical applications.

Scalability in manufacturing is defined as the capability to control process throughput without significant loss of manufacturing resources^26^. Putnik *et al.*^27^ reviewed scalability in manufacturing systems and discussed various definitions of scalability. In their work, they described scalability as the ability to upgrade or expand process capabilities whether quantitatively or qualitatively. Scalability is directly related to throughput and, thus, manufacturing costs. Because micro-/nanoscale fabrication processes usually have extremely low throughput and a precise process scale, scalability plays a more important role than in ‘conventional’ manufacturing. In this research, scalability is considered with respect to throughput and dimensional complexity. Similarly, expandability is considered as the ability to upgrade or expand process capabilities by bridging other processes.

For scalable manufacturing, micro-/nanomanufacturing systems have been developed with a view to bridging various processes, in line with trends in manufacturing generally. Integration and the bridging of different processes may enable the creation of synergies, while keeping the advantages of the individual processes, as shown Table 1. Manufacturing paradigms have changed to meet various customers’ requirements^28^—from conveyor production (1900s), flexible manufacturing (1980s), and reconfigurable manufacturing (1990s) to cloud-based manufacturing (2010s)^29,30^. Cloud-based design and manufacturing include concepts of distributed manufacturing, as well as adaptive process planning and set-up, to efficiently distribute and use manufacturing resources^31^.

Particularly at the nanoscale, due to the fundamental limits of process physics, fabrication techniques must be integrated to create synergies among the various processes. Qin *et al.*^32^ claimed that bridging gaps between ultra-precision machining and semiconductor processes was key for micromanufacturing; they also emphasized the importance of hybrid processes/equipment due to the limitations of individual processes, assisted by materials databases containing information about microstructures and size effects. Brousseau *et al.*^2^ also described a future trend leading to a top–down/bottom–up synthesis, as well as simultaneous optimization of process parameters and materials refinement. Putnik *et al.*^27^ claimed that a single element may be linked to other identical elements to raise scalability in performance or functionality, where the single element itself can also be scaled up or down. From that standpoint, the definition of a ‘hybrid’ process expands beyond the traditional definition. Traditionally, hybrid processes were considered to be simply a combination of multiple techniques acting simultaneously within the same processing area, such as laser-assisted machining^33,34^. Recently, this definition has been broadened to integrated configurations for creating synergies between more than two processes^35^; these processes are bridged within a standardized fabrication platform, with standardized data communication and modulation of the individual processes. To create synergies and develop a novel hybrid process, integrated hardware may or may not be required.

Following changes in manufacturing paradigms, many researchers have investigated micro-/nanoscale techniques for the manufacture of micro-/nanostructures with controllable geometry. Ok *et al.*^36^ applied photolithography techniques to roll-to-roll nanoimprint lithography, and created continuous and scalable pattering. Hilali *et al.*^37^ presented the scalable patterning of a 3D pyramid pattern using jet- and flash-imprint lithography^37^. Martìnez-Galera *et al.*^38^ controlled graphene’s electronic and optical properties by applying both bottom–up and top–down approaches. Salaita *et al.*^39^ used dip-pen nanolithography with 55 000 pen 2D array for fabrication of micropattern. However, from the perspectives of manufacturing, these micro-/nanomanufacturing processes typically are not yet able to produce free 3D complex structures, such as with undercuts of various angles, and also tend to be limited in terms of the materials that can be processed.

Several technologies that have been investigated within nanoscale techniques to fabricate complex 3D geometries are based on direct-writing methods^40^. Engstrom *et al.*^41^ reviewed various nanoscale additive processes and classified them in terms of process resolution and printing speed. Small tips can also be used for positioning and removing small amounts of material, as in dip-pen nanolithography^42^. Fischer *et al.*^43^ fabricated 3D freeform structure using ion implantation, silicon deposition, and selective silicon etching. However, these techniques usually focus on scalability in terms of process scale and geometric degrees of freedom rather than throughput.

Hybrid and bridging processes may enable significantly enhanced process capabilities and are expected to provide plausible solutions towards scalable nanomanufacturing. Although hybrid manufacturing still faces several challenges^44^, for example, hardware and processing costs, it is expected to provide breakthroughs toward scalable nanomanufacturing.

### CAD/CAM for micro-/nanomanufacturing systems

Following the development of micro-/nanomanufacturing technologies, CAD/CAM systems have been developed to assist in such fabrication processes. CAD/CAM systems have been adopted widely in manufacturing generally with the aim of assisting in precise, high-speed manufacturing^45^. Moreover, considerable research effort has focused on managing and improving process capabilities with standardized programming^46,47^; the nanoscale self-assembly system^48^ is one example.

In some instances, CAD/CAM has been applied to nanomanufacturing in simplified form^49^. Particularly for direct-writing technologies, CAD/CAM has been used to generate processing paths simply by changing the linear scale^50^. FIB processing is a representative example, because beam paths should be considered carefully. Since the ion beam has directional properties in material redeposition; thus, artifacts vary in terms of processing conditions and processing paths. Jamali *et al.*^51^ investigated different milling strategies in terms of different layering methods, and Lindsey *et al.*^52^ investigated controlling dose profiles to resolve the angular-dependent sputtering yield and redeposition problems.

Several software packages have been developed to assist in semiconductor processes^53^. Commercial software, such as INTELLICAD and MEMSCAP, can be used to design and simulate microdevice fabrication; these software packages are capable of constructing 3D structures based on the processes involved^13^.

However, existing CAD/CAM systems are not fully applicable to scalable nanomanufacturing. Flexibility and the expandability of individual processes have not been considered, because some systems were developed for specific processes. Furthermore, CAD/CAM for scalable nanomanufacturing requires not only nanoscale processing capabilities but also compensation for nanoscale effects^54,55^. A system should be able to assist and simulate the micro-/nanoscale processes involved. For effective and precise fabrication in scalable nanomanufacturing, it is important to establish a consensus for a novel CAD/CAM system.

## Results and discussion

### Requirements for scalable nanomanufacturing CAD/CAM systems

Based on the investigation in the section above, key characteristics required for CAD/CAM systems for scalable nanomanufacturing were discussed. Because scalable nanomanufacturing is still a developing area, some requirements are suggested based on the demands of various nanoscale processes. Furthermore, because various fabrication techniques can be used as sub-processes within a hybrid process, general characteristics were discussed. System details may vary in terms of specific sub-processes; nevertheless, these details are essential in providing basic insight into a novel CAD/CAM system.

Characteristics and requirements of scalable nanomanufacturing can be described as following: material manipulation and control at nanoscale^1,13,21,22,56–58^, combination of technologies^2,13,15,23,56,58^, integrated platform among many disciplines^2,13,21,22^, control of material properties over large areas^1,15,59^, and functional integration^2,15,23^. Understanding material behavior at nanoscale is the basic approach toward the scalable nanomanufacturing^21,58^. Brousseau *et al.*^2^ and Qin *et al.*^32^ emphasized the importance of hybrid processes/equipment which enable bridging gap between top–down or bottom–up synthesis. Tadigadapa and Najafi^13^ claimed the importance of integrated simulation with various engineering disciplines. Busnaina *et al.*^15^ claimed the importance of process control over large areas, that is, maintaining the necessary force at nanoscale. Furthermore, the importance of functional integration, that is, imaging, measurement, and manipulation, has been emphasized by many researchers^2,23^. From the perspectives mentioned above, the CAD/CAM systems require the following characteristics for scalable nanomanufacturing:

#### Characteristic 1 Process knowledge database

For expandable and precise fabrication, the system should have large process knowledge databases with respect to individual processes. Specifically, the process knowledge database should include error source analysis, size effects at the nanoscale, and uncertainties in the process.

When a process reaches the nanoscale, process knowledge plays a more significant role than in conventional scale fabrication. From the perspective of machine hardware, the contribution of each machine component to product precision varies in terms of the process scale. Thus, the error budget can vary with respect to process scale even on the same machine^60,61^; CAD/CAM systems should have information on such error sources and their influences.

CAD/CAM systems also need to have process knowledge at the nanoscale, because processes and materials at the nanoscale often show abnormal phenomena that simply do not occur at the macroscale. Many such phenomena are already known. For example, mechanical machining shows a different cutting mechanism when cutting is reduced to the nanoscale. Recent research has shown that ultra-precision machining (UPM) can achieve ductile machining even with a brittle material^62^. Moreover, crystal orientation has a significant influence on machined surfaces, due to the different mechanical properties in terms of direction^63^.

Another aspect that should be considered is the uncertainty of the process. In nanomanufacturing, it is important to consider uncertainty of process which may originate from machine tools and work material. These uncertainties can bring about difficulties in process control in terms of precision and accuracy. Representative examples are electron-/ion beam lithography and FIB milling. In lithography, a shot noise (dose fluctuation) limits a local position control of irradiated charged particles in the resist, resulting in a line edge roughness^64,65^. In FIB milling, although it can avoid the shot noise due to high ion dose during beam-matter interactions, it is difficult to forecast all nanoscale effects due to side effects such as secondary sputtering, surface swelling, material redeposition, and so on*.*^66^. Particularly in FIB milling, a grain (local microstructure) as a source of material’s randomness can play a role, so called ion channeling effect. The ion channeling effect can alter the material removal efficiency (sputtering yield) in the different grains^67^. Thus, simulation systems should have an appropriate knowledge database that accounts for uncertainty; as such, CAD/CAM systems for scalable nanomanufacturing will require a larger database among many disciplines than those for macroscale processes.

#### Characteristic 2 Standardized processing

Considering that manufacturing processes may be hybrid, the system should include various processes and be able to communicate information among those processes. From that standpoint, it is recommended that the system use open-source software for sharing design geometry and process planning.

CAD/CAM for scalable nanomanufacturing needs to be able to share information among different processes. Such communications should be performed via a standardized platform. Product geometry should be realized for the various processes; the process plan may be the simplest form of information to be transferred during the multiple steps of design and manufacturing. Similar to MEMS software, the system is required to reconstruct a 3D geometry-based process plan consisting of the processes involved. This requirement is also consistent with the requirements of cloud-based design and manufacturing^29^; individual processes may be considered as manufacturing-as-a-service. One characteristic of scalable nanomanufacturing is expandability; thus, it is recommended that the CAD/CAM systems use open-source software for ease of sharing information.

#### Characteristic 3 Active communication

To generate process plans efficiently, the system should be constructed based on a network, to provide communication between designers and process operators. Considering that nanomanufacturing can be achieved by integrating various manufacturing processes, process plans can vary in many ways. Communication between the designer and process operators has always been considered an important issue in manufacturing. Traditionally, design has been constrained by manufacturing capabilities; thus, designers should consider processes for effective manufacturing^68^. This paradigm is referred to as design for manufacturing (DFM).

Recently, however, the manufacturing paradigm has shifted towards realizing design by expanding the available process capabilities^69,70^. This new paradigm is referred to as manufacturing for design (MFD). Design constraints sometimes become disadvantages in the product; application of novel manufacturing processes may, directly or indirectly, contribute to improving the final product by changing the process or design^71^. For example, additive manufacturing can contribute to reducing the weight of aerospace products by removing unnecessary parts that were required only for a ‘conventional’ metal-casting process^72^.

Similar to this paradigm shift from DFM to MFD, both part design and process planning can vary in terms of process capabilities within scalable nanomanufacturing. Part design can be changed with respect to process capabilities, and fabrication processes can be simplified with respect to operator proficiency. In some nanoscale CAD/CAM systems, part design is correlated with the fabrication process^73^; however, design and process plans need to be freely editable to minimize limitations on part geometry.

To address this, a CAD/CAM system should feature active communications between designers and process operators. However, even with the help of knowledge databases, designers may not be familiar with fabrication processes, and operators may not understand designers’ intentions. For efficient design modification and process plan generation, designers and operators should be able to access design and process plans simultaneously, for more productive discussions regarding manufacturing.

Thus, CAD/CAM systems should be constructed on an active communications network. Additionally, part design should be able to be modified during and/or at the end of the design, as well as correlated with the process plan. The correlation between the design and process does not need to be forced so as not to limit part design.

#### Characteristic 4 Adaptive interpolation

For nanoscale fabrication, a CAD/CAM system should be able to handle nanoscale components in design and manufacturing. The system should cope with fundamental building block, that is, single atomic layer, and also cope with nanoscale tool path as well as tool contact point management.

In particular, nanoscale fabrication requires much careful tool path planning due to required tolerances, number of numerical control (NC) programming lines, and the system delay of the machine. A tool path generated by conventional CAM system always includes an error due to interpolation of curves, but the error usually stays within the tolerance that conventional manufacturing generally specifies and accepts^74^. However, at nanoscale fabrication, there are two factors to be considered in tool path generation; the number of digits and segmentation. The number of digits to be handled by process planning, such as NC code, below the decimal is five, six, or seven as many of nanoscale level machines have 10 or 1 nm command resolution. Recent advancement of machine tool technology even demonstrated 1 Å command resolution^75,76^. It is not a matter of simply adding additional digits in the computation, but careful consideration of truncation error during interpolations.

The segmentation (interpolation scheme) also significantly influences deviation error of the tool path. At nanoscale, much smaller segmentation or precision fitting of the curve is required to reduce the deviation error. The sum of truncation error and deviation error imposed by computation and interpolation should be at least one order of magnitude smaller than conventionally accepted tolerances at nanoscale fabrication practices, considering additional error that could be caused by other sources, such as the positioning accuracy of the machine tools^77,78^.

An additional critical consideration for CAM processing at nanoscale is proper time allocation for each NC code line. The machine controller needs to process NC code in order to drive the machine, and processing time and delay by the machine drive system should be simultaneously synchronized for precision control of the system. In most cases, the mentioned tolerance requirement means more segmentation of lines which in turn results in a huge amount of NC code lines. The cycle time of each NC code line is extremely short and sometimes less than the time of the controller process and delay of the drive system. If this happens, the subsequent NC code lines will not be processed, which eventually could cause overflow error of the processor or other critical error.

Therefore, the minimum cycle time for each NC code line should be considered for designing of an interpolation scheme at nanoscale in CAM system. In some cases, the processing accuracy of the machine is not determined by the machine accuracy but by inherit system delay between the controller and the machine drive system, which has not been an issue in conventional scale. If this is the case, the speed of the process (either feed or speed) should be set slower in consideration of system delay and CAM should be able to identify this issue during calculation.

The same discussion should be applied to the CAD system. When a CAD model for a specific part requires nanoscale precision, all the arguments mentioned in the previous two paragraphs for CAM system should be carefully considered. At nanoscale fabrication, a CAD/CAM system should conduct detailed and thorough error budgeting analysis with actual physical system level verification.

#### Characteristic 5 Consistent coordinate system

For precise fabrication, a CAD/CAM system should be able to indicate the same work coordinates consistently during fabrication. Scalable nanomanufacturing can consist of multiple manufacturing processes with different environments and process scales. However, the work coordinates should be maintained consistently.

Fixturing, or setting up precise work coordinates, is one of the most challenging issues in nanomanufacturing^79^. Because the error budget varies widely in terms of process scale, CAD/CAM systems should have information on work coordinate setting and predicted errors. Particularly in hybrid processes, simply moving the sample can have a large influence on final product precision, because the fixturing set-up cannot be maintained between steps. Various methods have been investigated for coordinate references, and they have pros and cons. An optical reference pattern is commonly used in MEMS/NEMS fabrication; however, the precision is limited by the wavelength^80^.

Particularly in scalable nanomanufacturing, having consistent coordinates during fabrication is important because each process has a different processing environment and process scale. A reference pattern may consist of multiple steps in which each pattern has a specific precision with respect to the related fabrication process. Each step can also have different physics within a different process environment.

Thus, a CAD/CAM system should have information on how to set-up work coordinates for the various processes involved and have a consistent coordinate system during the multiple steps of a process. Information on coordinate set-up can include reference mark information, use of additional peripheral devices for zeroing axes, and predicted errors from the reference pattern used.

#### Characteristic 6 Management of peripheral devices

To use manufacturing resources fully, a CAD/CAM system should also have information on peripheral devices associated with individual processes. When process scale is reduced, the importance of peripheral devices increases to provide more precision. Because a scalable nanomanufacturing process may consist of various micro-/nanoscale fabrication steps, the system will have a larger number of peripheral devices versus a conventional fabrication process.

Manufacturing systems typically require more peripheral devices as the process scale decreases; more resources and controls are required to perform nanomanufacturing. This trend can be observed easily in terms of energy consumption; the proportion of pure processing energy decreases as the process scale decreases^81–83^. This means that additional energy is required at the nanoscale to support the main process. For example, peripheral devices are usually used to provide a specific process environment, to set work coordinates, and to measure processed geometry. For an effective process, these peripheral devices should be managed appropriately and should be able to assist each other.

Thus, the CAD/CAM system should have information on peripheral devices, and should also be able to manage those devices with respect to the desired function. Management of peripheral devices can contribute not only to using machine resources but also to improving the capabilities of each process.

Table 2 lists the requirements for CAD/CAM systems for scalable nanomanufacturing. Representative characteristics include process knowledge databases, standardized processing, active communications, adaptive interpolation, a consistent coordinate system, and the management of peripheral devices. Each requirement corresponds with characteristics of scalable nanomanufacturing, that is, process knowledge database—material manipulation and control at nanoscale.

Impacts of each requirement can be discussed with respect to the corresponding characteristics. Process knowledge database is directly related to material manipulation and control at nanoscale; it is an essential requirement to achieve precision at nanoscale. Without standardized processing, 3D geometry and process information cannot be reconstructed, hence different processes are not able to be fully integrated with each other. Active communication is important to improve the process efficiency, because it promotes communications between users and operators, as well as communication among various disciplines. Adaptive interpolation is essential to control material properties over large areas within a certain scale. Consistent coordinate system and management of peripheral devices are related to efficiency and sustainability. Without management of peripheral devices, manufacturing and maintenance costs will significantly increase.

Some of these requirements are also requirements for hybrid manufacturing, and some are for cloud-based design and manufacturing. Because scalable nanomanufacturing is a state-of-the-art technology within manufacturing processes, some requirements are shared by other recent manufacturing paradigms. Nevertheless, some requirements are unique and apply only to scalable nanomanufacturing. It is expected that the characteristics listed will boost the process capabilities in scalable nanomanufacturing and contribute to new manufacturing processes.

### Comparisons with conventional CAD/CAM systems

Although some characteristics are shared with other manufacturing paradigms, the suggested characteristics can also differ from those for conventional CAD/CAM systems. The differences are due to not only the process scales but also knowledge databases and expandability.

Although the system should have active communication between designers and operators, this can still be distinguished from a conventional web-based CAD/CAM system. In the 1990s and 2000s, various web- and agent-based CAD/CAM systems were developed for distributed manufacturing^84,85^. However, the network system simply provided capabilities in terms of design, process planning, and process simulation with respect to programmed manufacturing rules^86–88^. Systems were able to distribute resources in CAD/CAPP/CAM^89^, rather than considering flexibility and expandability based on nanoscale knowledge.

However, scalable nanomanufacturing is not limited simply to distributed manufacturing, as it requires active communication from both designers and process operators. Based on the MFD paradigm, part design is not constrained by currently involved processes. Additionally, design and process plans can be accessed readily and modified with respect to various conditions, that is, processes or operator proficiency.

By listing key requirements, this research provides insight on CAD/CAM systems of scalable nanomanufacturing. Though existing tools may include some characteristics^13,53,54^ among the mentioned requirements, the suggested CAD/CAM systems concept includes a wider range of manufacturing in terms of scalability (throughput and dimensional complexity). By considering expandability, process can be more easily accessible, or can be improved with hybrid or bridging. An example scenario of a network-based system is shown for hybrid 3D printing in the next section.

### Network-based system for hybrid 3D printing scenario

#### Nanoscale 3D printing system

Towards scalable nanomanufacturing, we developed a hybrid 3D printing process by integrating various micro-/nanoscale processes^20,90,91^. The process was developed for freeform 3D printing at the nanoscale with multi-material capabilities, by integrating AFN printing (denoting aerodynamically focused nanoparticle printing), micromachining, FIB, and spin-coating processes.

**Figure 1:**
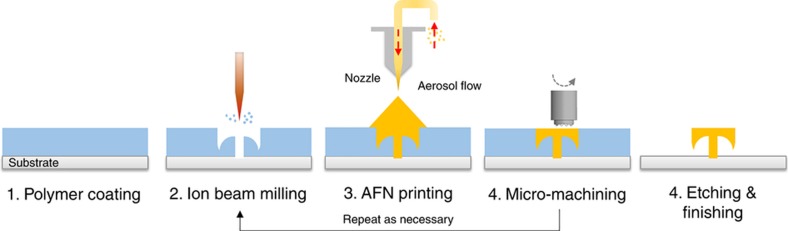
Process flow chart (improved based on data from Ref. 90 with kind permission from Elsevier).

Figure 1 shows a process flow chart of the system developed. A spin-coating process was used to create a sacrificial layer. FIB milling was then applied to create a 3D pocket on the polymer layer. Nanoparticles were then printed onto the pocket, and micromachining was performed for localized planarization. Wet etching and finishing resulted in 3D printed structures. Because AFN printing can print metal/ceramic inorganic materials, structures can be fabricated with various functional materials^92^.

The three main processes, AFN printing (additive), micromachining (assistive), and FIB (subtractive), have different process environments and scales. AFN printing is a dry, room-temperature nanoparticle printing process, and has a process scale width of 30 μm. In contrast, FIB requires a high level of vacuum and has process scales that vary from 50 nm to tens of microns. Micromachining is performed under dry, room-temperature conditions and was designed for a tool diameter of 30 μm; however, it can operate at a significantly higher processing rate than the other two processes.

For efficient bridging, individual processes were investigated to improve the compatibility. Printing strategies were developed to reduce the printing scale^20^, and different tool geometries were evaluated to improve the cutting performances^93^. Various FIB paths were suggested to minimize directional artifacts of scanning beam^94^. Further, alignment patterns with multiple marks were designed and implemented to keep the same coordinate system among different processes.

**Figure 2:**
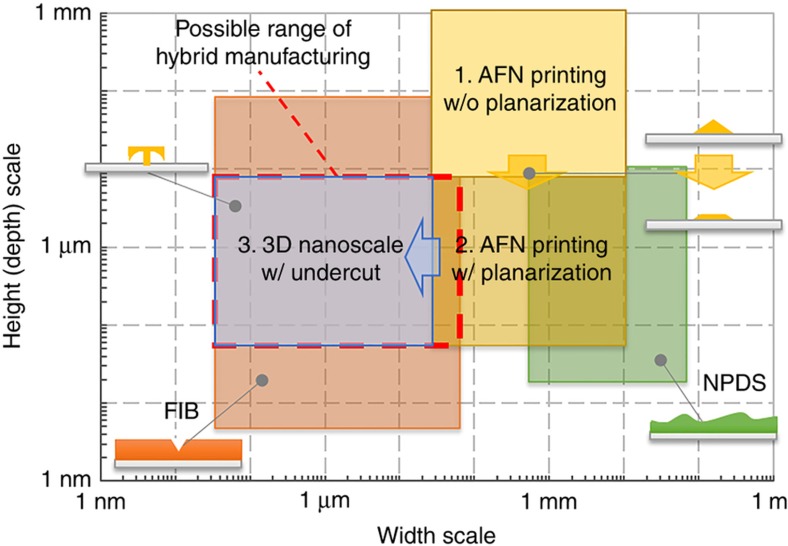
Width and height scale of the developed hybrid process. 3D: three-dimensional; AFN printing: aerodynamically focused nanoparticle printing; FIB: focused ion beam; NPDS: nanoparticle deposition system. (reproduced based on data from Ref. 90 with kind permission from Elsevier).

By bridging these processes, process scales can be improved beyond that of each individual process. Figure 2 shows process scales of the hybrid 3D printing process and that of each individual process. With the assistance of the planarization and sacrificial layer, the process width scale can be reduced to 50 nm, and the height scale can be reduced to hundreds of nanometers. Furthermore, the process developed can fabricate free 3D structures with undercut, using any inorganic metal ceramic material. More detailed descriptions of process development are discussed in recent publications^20,90,91^.

The hybrid 3D nanoprinting process has advantages in terms of process scale, geometric degrees of freedom, and material selectivity versus other processes. This process has wider material selectivity than beam-based nanomanufacturing, smaller process scale than aerosol-based nanomanufacturing, and higher geometric degrees of freedom than tip-based nanomanufacturing.

From the perspective of manufacturing *per se*, the hybrid 3D nanoprinting process is not yet appropriate for mass manufacturing; however, it can be applied to mass manufacturing by playing a role similar to that of rapid tooling in an additive process. In terms of throughput, FIB milling is the most time-consuming process, due to its low material processing rate. However, if shaping on a coated polymer could be substituted by other processes (that is, embossing), the suggested process can be applied to the mass manufacturing; the process width scale of nanoparticle printing and micromachining can be extended up to several millimeters, and their throughput is already high enough (as shown in Figures 1 and 2). By fabricating molds/tools for mass manufacturing, the proposed process can contribute to nanomanufacturing, without losing advantages in multi-material capability (metal/ceramic inorganic materials). Furthermore, the process adopts different fabrication methods and creates synergies from the individual processes, without complicated, multi-step hardware integration. Thus, the hybrid 3D nanoprinting system developed can be considered as a mass nanomanufacturing example. With the integration of patterning technologies, such as nanoimprint lithography or hot embossing, the system can be improved for scalable nanomanufacturing in terms of throughput.

#### Network-based CAD/CAM system

During process development, the CAD/CAM system was also considered and developed. Because the hybrid process consisted of four individual processes, a CAD/CAM system was required for cooperative control and management. The main aim with the CAD/CAM system is to generate a process plan for the different processes.

Various attempts have been made to develop CAD/CAM systems that consider the requirements mentioned above. However, with regard to scalable nanomanufacturing, these requirements would be difficult to implement with a conventional CAD/CAM system. As such, novel software was developed to address this issue: the software was based on both process-oriented architecture and design-oriented architecture. Through several attempts^95^, considering the requirements for scalable nanomanufacturing listed, a novel system was developed to enable active communication between designers and process operators. One of former CAD/CAM system was of a stand-alone type with automatic process planning, and it was not easy to deliver efficient process plans even with simple geometry.

The final CAD/CAM system was constructed on the web (http://hccl.snu.ac.kr/webcad), based on the OpenJSCAD (ver.0.018, 10/5/2014 release) platform^96^. The most significant difference from a CAD/CAM system for conventional scales was that the system was developed based on a network to promote communication between users and process operators. Its use with Google Chrome is recommended.

**Figure 3:**
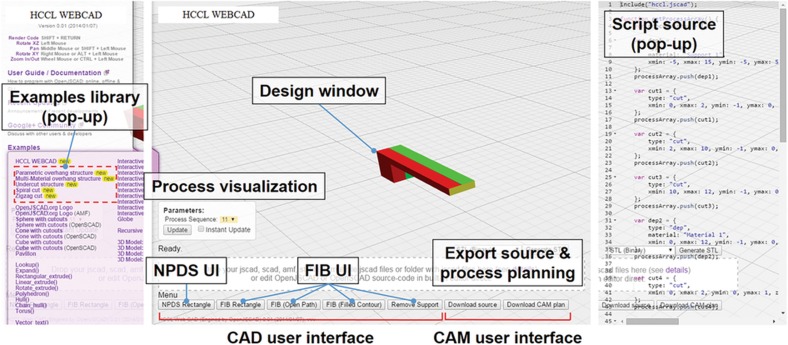
Screenshot image and UIs of the CAD/CAM system developed. Example library provides representative geometry examples. CAD/CAM: computer-aided design/manufacturing; UI: user interface.

Figure 3 shows a screenshot image of the CAD/CAM system developed. At the bottom of the page, nanoparticle deposition system (NPDS)^97^ and FIB user interfaces (UIs) are available, enabling users to create any 3D design, based on a layer-by-layer process, with geometric and material information; notably NPDS includes dry nanoparticle printing processes, for example, AFN printing. The design can be performed using both a UI and script code. Because the UI directly generates the corresponding script code, part design and process plans can be modified readily using the script code window. Since the geometry information is stored in the script code, it can be directly shared and re-generated with other manufacturing process. Micromachining is automatically implemented after the NPDS process; however, some planarization can be removed, as desired.

Any 2.5D structure can be profiled using FIB UIs, from rectangle to freeform contours. Additionally, the system provides representative examples, such as a multi-material cantilever, a microcapacitor, or a resistor, as a library on the left menu. Thus, users can directly create simple, common structures by controlling the geometric parameters. Some representative examples are shown in recent publications^20,91^.

Then, the system provides CAM files that can be used for CAM of each process, as well as overall macro process planning. Each process has its own CAM system. Operators can fabricate parts following the overall process planning, by applying delivered CAM files to each process. For the nanoparticle printing and micromachining processes, customized LabVIEW programs were used, and CAM files include process parameters and tool paths. For the FIB milling, a pattern generator (ELPHY Quantum, RAITH GmbH, Germany) was used, and the delivered CAM file includes beam processing conditions and its path.

The system also provides design and manufacturing guidelines for designers and operators by giving warnings based on DFM rules. Most rules are due to process limitations, such as material compatibility between the bottom and top layers printed^98^. Also, the printed geometry is limited by the process, so the system recommends proper design/fabrication methods, similar to FIB path-generation strategies.

**Figure 4:**
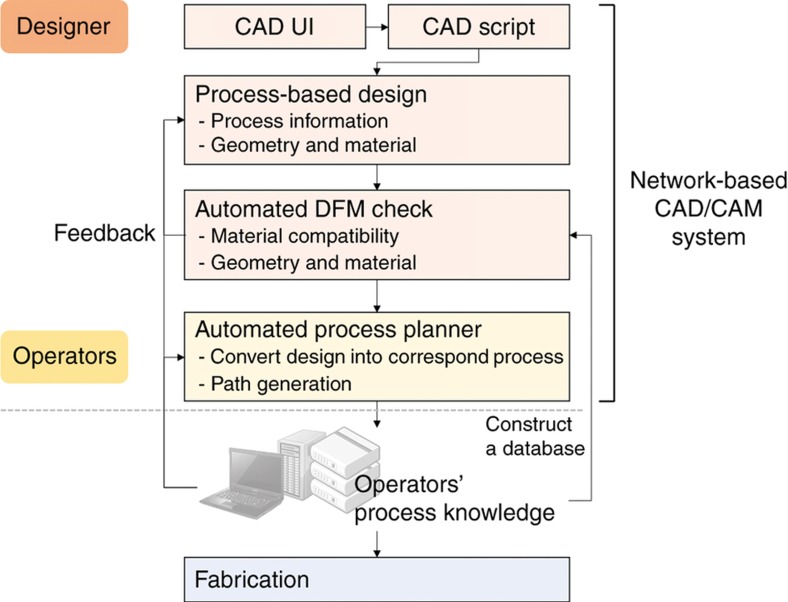
Schematic diagram of the network-based CAD/CAM system. DFM: design for manufacturing.

Figure 4 shows a schematic diagram of the CAD/CAM system developed. Designers can design a part based on the fabrication process, and the DFM rules check the manufacturability automatically at the design stage. A process plan can be generated both automatically and by process operators. Also, both designers and operators can check the automatically generated process plan; this can be revised with respect to the operators’ proficiency.

Table 3 shows a comparison of the requirements for a CAD/CAM for scalable nanomanufacturing and the characteristics of the CAD/CAM system developed. As mentioned so far, characteristics of the CAD/CAM system developed are coupled with the suggested requirements, that is, process knowledge database—DFM rules warning system. However, peripheral devices are not currently managed by the CAD/CAM system. Each process has its own peripherals, and the CAD/CAM system provides CAM files per each individual process. For more efficient bridging, peripheral information will be recorded and managed by the CAD/CAM system. Peripherals may include several modules, simply from vacuum pumps to manipulation/measurement modules in the FIB chamber. These peripheral modules can contribute not only to the improvement of individual process efficiency, but also to creation of novel processes, that is, scribing or assembly. Peripheral information will be shared both with designers and operators. Nevertheless, the current developed system has a process knowledge database in DFM rule form, standardized processing, active communication, and a consistent coordinate system. The system provides an open platform for hybrid 3D printing between designers and operators. Both design and process plans can be modified and visualized easily. In addition, DFM rules will be kept updated with more experimental data. New processes may be updated with correspondent CAM systems. We anticipate this approach can contribute to the development of a CAD/CAM system for scalable nanomanufacturing.

**Figure 5:**
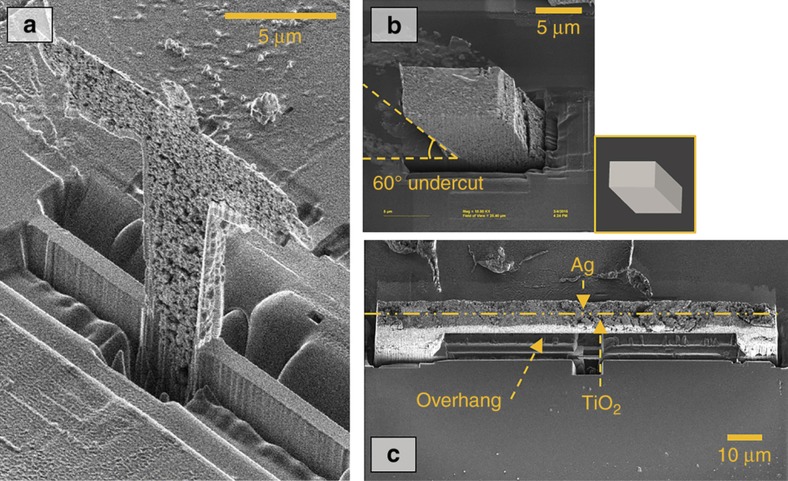
Examples fabricated using the hybrid three-dimensional (3D) printing process at the nanoscale: (**a**) 3D printed micro-Jesus statue^20,91^, (**b**) tilted pillar structure with a tilt angle of 60°(Ref. 20), and (**c**) bimaterial cantilever (reproduced based on data from Ref. 90 with kind permission from Elsevier).

Figure 5 shows examples fabricated using the hybrid 3D printing system developed. Detailed descriptions of the fabrication process are provided in recent publications^20,90,91^. Figure 5a shows a statue with nanoscale features and various types of undercut. Figure 5b shows a tilted pillar with an undercut of 60°, and Figure 5c shows a bimaterial cantilever. The items in Figures 5a and b were made of silver, while that in Figure 5c was made of silver and titanium oxide. These fabricated examples show 3D printing at the nanoscale and hybrid characteristics.

As mentioned before, the process can be applied in mass manufacturing by fabricating a mold or tool within a mass manufacturing process. Once the shape has been settled, FIB milling can be substituted, and throughput can be significantly improved to be applied to the mass manufacturing. One of the current issue is filling of nanoparticles to the desired pocket. Sometimes structures have large pores due to unstable variations of the printing process. Another issue is the deformation control of polymer layers during a mold or tool fabrication at micro-/nanoscale. Precise adaptive control with more process knowledge would be required in order to overcome these problems. Nevertheless, by combining it with other mass manufacturing techniques at the nanoscale, the process can be used to fabricate 3D freeform structures with improved throughput. From this perspective, the network-based CAD/CAM system can contribute to efficient bridging of different disciplines and communication with each other. Though mentioned processes have various environments (that is, different vacuum conditions), nanoscale 3D printing process can be constructed in a much more beneficial way than one hardware configuration. As mentioned so far, bridging and hybrid are considered as key requirements in scalable nanomanufacturing due to the fundamental limits of individual process physics. A network-based CAD/CAM system can provide a platform of bridging with the suggested key requirements. The structure and CAD/CAM system can thus advance scalable nanomanufacturing research efforts.

## Conclusions

In this paper, micro-/nanoscale fabrication techniques were briefly reviewed, and key requirements for a CAD/CAM system for scalable nanomanufacturing were listed. Among various fabrication techniques, bridging different processes is considered to be a breakthrough in going beyond existing fundamental limits for each process alone. CAD/CAM systems for scalable nanomanufacturing should have the following key characteristics: (1) process knowledge database, (2) standardized processing, (3) active communication, (4) adaptive interpolation, (5) a consistent coordinate system, and (6) management of peripheral devices. These characteristics and requirements are expected to contribute to the development of a novel manufacturing paradigm.

As an example, we describe a novel CAD/CAM system for hybrid 3D printing at the nanoscale. The system was constructed on the network-based OpenJSCAD platform for active communication among designers and process operators. Characteristics of the developed CAD/CAM system and suggested key requirements were compared. This network-based 3D printing system with key requirements can contribute to the paradigm shift from DFM to MFD. Design and manufacturing plans can be controlled flexibly and adjusted. 3D printed features at the nanoscale were shown in the fabricated samples. Because scalable nanomanufacturing is one of the most advanced manufacturing techniques, characteristics of CAD/CAM systems can also be applied to the novel concept of hybrid processes. Considering that bridging different processes may provide breakthroughs for a future manufacturing paradigm, it is also suitable for cloud-based design and manufacturing.

Through this research, the concept of CAD/CAM for scalable nanomanufacturing has been discussed and developed. Scalable nanomanufacturing will enable variable engineering applications with the assistance of a novel CAD/CAM system. We anticipate that this approach will contribute to the development of CAD/CAM for scalable nanomanufacturing, as well as a wide range of hybrid processes.

## Figures and Tables

**Table 1:** Examples of micro-/nanoscale fabrication techniques for three-dimensional structures^20–25^

	Process scale	Resolution	Geometric degree of freedom	Throughput	Process materials
Energy beam-based (FIB-chemical vapor deposition,* *and so on)	***	**	***	**	**
Liquid/aerosol-based (Electro-hydrodynamic-jet, ink-jet, and so on)	**	*	**	**	***
Tip-based (Dip-pen nanolithography, and so on)	*	***	**	*	***
Imprinting/molding (Nanoimprint, hot embossing, and so on)	***	***	*	***	*
Machining (Ultra-precision machining, and so on)	**	**	***	**	***
Hybrid	***	***	***	***	***

***—good, **—average, *—weak.

**Table 2:** Requirements for CAD/CAM systems for scalable nanomanufacturing

RQMTS No.	Details
1	**Process knowledge database** Large knowledge databases, including concepts of error source analysis, size effect, and uncertainties in the fabrication process.
2	**Standardized processing/open-source programming** Sharing information on part geometry and process planning as well as process information.
3	**Active communication** Providing communication between designers and process operators to edit and improve part geometry and process planning.
4	**Adaptive interpolation** Interpolating coordinates within the range of volumetric error of the machine and providing sufficient computational resources.
5	**Consistent coordinate system** Providing information on setting work coordinates and predicted errors during the multiple steps of fabrication processes.
6	**Management of peripheral devices** Managing peripheral devices to use machine resources fully and to improve process capabilities.

**Table 3:** Comparison of requirements and the CAD/CAM system developed

RQMTS No.	Details	CAD/CAM system
1	Process knowledge database	DFM rules for individual processes were implemented in the system, with caution messages to designers.
2	Standardized processing/ open-source programming	The system was constructed using OpenJSCAD platform. Part geometry/process plans can be exported in text format.
3	Active communication	The system was constructed on a network, and designers and operators can access and modify design/process plans.
4	Adaptive interpolation	CAM files were generated for each individual process.
5	Consistent coordinate system	Alignment patterns for three different processes were implemented in the hybrid process.
6	Management of peripheral devices	—

Abbreviations: CAD/CAM, computer-aided design/manufacturing; DFM, design for manufacturing.
